# Physicochemical and hygienic effects of *Lactobacillus acidophilus *in Iranian white cheese

**Published:** 2012

**Authors:** Razzaqh Mahmoudi, Hossein Tajik, Ali Ehsani, Payman Zare

**Affiliations:** 1 *Department of Food Hygiene and Aquatics, Faculty of Veterinary Medicine, University of Tabriz, Tabriz, Iran; *; 2 *Department of Food Hygiene and Quality Control, Faculty of Veterinary Medicine, Urmia University, Urmia, Iran; *; 3 *Department of Pathobiology, Faculty of Veterinary Medicine, University of Tabriz, Tabriz, Iran. *

**Keywords:** Iranian white cheese, *Listeria monocytogenes*, Probiotic, Starter

## Abstract

Increasing incidence of food-borne disease along with its social and economic consequences have led to conducting extensive research in order to produce safer food and develop new antimicrobial agents; among them, extensive use of probiotics and bacteriocins as biological additives is of significant importance. The aim of the present study was to evaluate the interactions (growth behavior and survival) of *Listeria monocytogenes* and *Lactobacillus acidophilus* in various stages of production, ripening and storage of Iranian white cheese. Changes in pH values at different stages of cheese ripening, along with changes in organoleptic properties of cheese were also assessed. Compared to other treatments, in the treatment of cheese with probiotic agent without starter, the most significant decrease in *Listeria monocytogenes* count at the end of ripening stage was observed (3.16 *Log* per gram cheese compared with the control group) (*p *< 0.05). Survival of probiotic bacteria in control samples of cheese were significantly higher when compared to cheese sample contaminated with *Listeria* (*p *< 0.05). White probiotic cheese with starter had the highest of sensory acceptability (*p *< 0.05). *Listeria Monocytogenes* count decreased during ripening period of probiotic white cheese but the bacteria survived in probiotic white cheese. *Lactobacillus acidophilus* count decreased during ripening period of white cheese but it did not lower to less than 10^6 ^CFU per g at the end of ripening and storage periods.

## Introduction

*Listeria monocytogenes* is a foodborne pathogen and toxicities caused by it mostly occur due to consuming milk and its products.^1^ In USA, *Listeriosis *accounts for more than 25% of mortality due to foodborne diseases and in this regard, milk and its products are of significant importance.^2^ Extensive spread in environment, ability to grow in a wide range of pH and refrigerator conditions and tolerating high amounts of salt lead to the fact that this bacterium is a very dangerous pathogen in food industries. Concerns for safety of some chemical preservatives have led to increased inclination towards natural preservative compounds with microbial and herbal origins.^1^ In this regard, different strategies have been suggested for control of this pathogen in dairy industry, among them using probiotic agents and bacteriocins as biological additives has been extensively studied.^3^

Lactic acid bacteria and *Bifidobacteria *are the most common probiotic agents being used in dairy products. Resistance to acid conditions of stomach, bactericidal activity of bile salts and production of lactic acid lead to their survival in gastrointestinal system. Among the potential advantages of Functional Foods for human health the following can be noted: improvement of gastro-intestinal microflora balance, preventing growth of pathogenic microorganisms, enhancement of immune system, relieving digestive disorders, improvement of irritable bowel syndrome. Antimicrobial activity of probiotic bacteria has led to their extensive use in producing functional foods in order to enhance consumers’ health.^4^ Lactic acid bacteria producing bacteriocins are part of digestive system microflora and play very significant role in host resistance. These microorganisms prevent growth and colonization of pathogenic organisms in digestive system by competing for adhering sites in digestive system, competing for the intake of nutrients and producing antimicrobial compounds (for example bacteriocins and short chain fatty acids)^5^. Lactic acid bacteria producing bacteriocins (including *Lactobacillus acidophilus* and other species of *Lactobacilli*) and bacteriocins can be used as biological preservatives in order to inhibit growth of *L. monocytogenes* in various kinds of foods especially fermentative dairy products like yogurt and cheese.^6^^,^^7^ The purposes of this study were to assess the growth of *L. monocytogenes*, survival of *L. acidophilus* in various stages of cheese ripening and storage, and pH changes along with changes in organoleptic properties of cheese.

## Materials and Methods

**Test Microorganisms. **Lyophilized culture of *L. monocytogenes* ATCC 19118 was obtained from the culture collection of the Department of Microbiology, Faculty of Veterinary Medicine, University of Tehran (Tehran, Iran). Subcultivation and preparation of inocula, was conducted according to standard method.^8^ Commercial mesophilic cultures of *Lactococcus lactis* subsp. *lactis* and *Lactococcus lactis* subsp. *cremoris* were obtained from Chr. Hansen A/S (Denmark) and used as direct vat-starter cultures. A commercial lyophilized culture of the probiotic* Lactobacillus acidophilus* ATCC 4356 was obtained from the Iranian Organization of Industrial Research. Sub-cultivation and preparation of probiotic bacteria were conducted according to the standard method^6^*.*

**Manufacturing and sampling of Iranian white** **cheese. **Iranian white brined cheese was made according to the following procedure: cow’s milk (2.5% fat) was pasteurized (72 ˚C for 16 Sec) and placed into a small stainless steel cheese container fixed within a larger pilot-plant-sized, steam-jacketed cheese vat partly filled with water. After warming the milk to 35 ˚C, it was inoculated with the test organism at 3 10^3 ^CFU per mL in separate groups. Next, 0.5% (v/v) starter culture *L. lactis* subsp. *lactis* and *L. lactis* subsp. *cremoris*, (Chr. Hansen A/S, R 704, Denmark) and probiotic bacteria (*L.* *acidophilus*, 10^8^-10^9^ CFU per mL) were added. Thirty minutes later, the pH of the milk was reduced slightly (5.4- 5.6) and 2 mL of microbial rennet (Meito, Sangyo Co., Japan) was added accompanied by 0.2 µg mL^-1^ CaCl_2_. One hour after adding rennet, the resulting coagulum was cut, and the cut curd was transferred into rectangular metal hoops (28×12×12 cm^3^) and allowed to drain for 6 hour. After draining at room temperature (22 ˚C), the cheese was cut into pieces (12×8×6 cm^3^) and placed into 20% sterile salt brine for 8h at 22 ˚C. The brine was then removed and cheese pieces were placed into sterile containers and covered with 8% sterile salt brine. Cheese in this brine was ripened at 14˚C for 15 days, and then stored at 4 ˚C for 45 days.^9^ Cheese samples for enumeration of *L. monocytogenes* was taken following times: 0, 1 and 7 hours, and then 1, 7, 15, 30, 45, and 60 days, and *L. acidophilus *were taken at days 1, 7, 15, 30, 45, and 60. All procedure was also carried out for the preparation of uninoculated (without *L. monocytogenes*) cheese, which was used for sensory evaluation. 

**Bacterial enumeration.** From different sections of the cheeses, 10 g samples were pooled in 90 mL of sterile 0.1% (w/v) peptone water (Merck, KGaA, Darmstadt, Germany) in sterile 500 mL stomacher bags. Samples were blended in a Stomacher 400 (Interscience*,* Saint-Nom-La-Breteche, France) for 3 min. *L. monocytogenes* counts were determined on PALCAM *Listeria* agar (Merck, Darmstadt, Germany) with PALCAM *Listeria* selective supplement (Merck, Darmstadt, Germany) after incubation at 37 ˚C for 48h. MRS agar (Merck, KGaA, and Darmstadt, Germany) was used for the enumeration of *L. acidophilus.*

**Changes in pH values. **Using a pH meter (CORNING model 220) equipped with a flat-bottomed standard combination electrode, pH values were conducted during ripening and storage of the cheese at each of the following days: 1, 7, 15, 30, 45, and 60.

**Sensory evaluation** **. **The sensory effects of adding probiotic bacteria to Iranian white cheese were evaluated using an acceptance test. White brined cheese samples with *L. acidophilus* and starter culture were equally divided into seven parts of 500 g each and placed on white plates coded with three-digit random numbers. The sensory evaluation was performed by a panel of seven judges consisting of the scientific staff of the Department of Food Hygiene, Faculty of Veterinary Medicine, Urmia University experienced in the sensory analysis of food. Water was provided for mouth washing between samples. Each panelist evaluated the samples by rating them using a nine-point scale, where 9 = like extremely, 8 = like very much, 7 = like moderately, 6 = like slightly, 5 = neither like nor dislike, 4 = dislike slightly, 3 = dislike moderately, 2 = dislike very much and 1 = dislike extremely, for various characteristics such as (appearance) color, odor and flavor.^10^

**Statistical analysis** **. **The variability of acceptance or liking of the samples was analyzed by ANOVA and Fisher's Least-Significant-Difference procedure (LSD), using SPSS for Windows Version 17.0 (SPSS Inc., Chicago, IL, USA). All experiments were conducted in triplicate. Data related to the mean values of microbial counts and physicochemical evaluations were subjected to analysis of variance (ANOVA). Results were considered statically significant when *p* < 0.05.

## Results

**Fig. 1:**
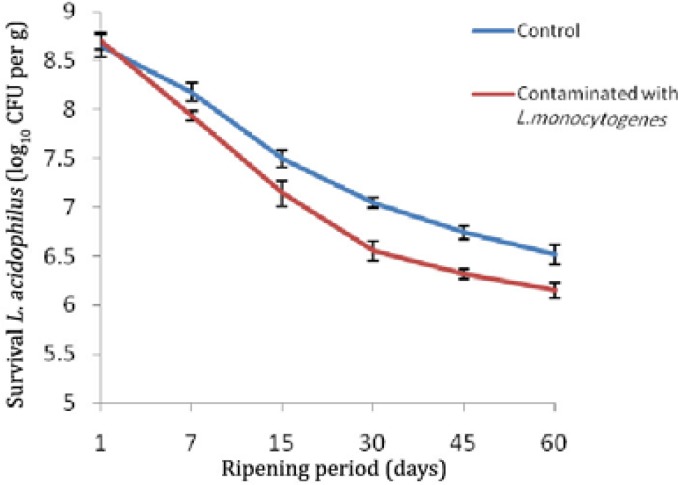
Durability *L. acidophilus *during ripening period of Iranian white cheese (Control: with probiotic agent and without *Listeria*).

Evaluation of the *L. monocytogenes* count in all treatments during ripening period was shown in Table 1. In treatment of cheese with probiotic bacteria without starter, the highest decrease count was observed compared with other treatments (*p *< 0.05). In this treatment, decrease in *L. monocytogenes* count at the end of ripening period of Iranian probiotic white cheese was 3.16 Log per gram of cheese higher than compared with control group, while in treatments of cheese with starter and probiotic bacteria and cheese with starter, decreases in *L. monocytogenes* bacterial count higher than compared with control treatment were 2.36 Log and 1.81 Log, respectively. Durability of *L. acidophilus* in all treatments during various stages of ripening Iranian white cheese was evaluated (Fig. 1). Survival rate of *L. acidophilus* in control sample of cheese was higher compared to sample of cheese contaminated with *L. monocytogenes* (*p *< 0.05).

**Table 1:** Growth response of *L. monocytogenes* affected by probiotic, starter, and their combinations in Iranian white cheese over a 60-day storage period.

**P**	**S**	**log**_10_ ** (CFU ** **per ** **mL or g) ± SD on sampling days**							
		1 (d)			7 (d)	15 (d)	30 (d)	45 (d)	60 (d)
		0 (h)	1 (h)	7 (h)					
–	–	3.00 ± 0.06^a^	4.95 ± 0.05^b^	5.72 ± 0.02^c^	5.52 ± 0.02 ^c^	6.84 ± 0.04^d^	7.49 ± 0.02 ^e^	7.80 ± 0.09 ^e^	7.81 ± 0.04^e^
–	+	3.04 ± 0.04 ^a^	3.42 ± 0.07 ^a^	4.22 ± 0.07 ^b^	4.93 ± 0.03 ^b^	5.45 ± 0.05^c^	6.37 ± 0.03 ^d^	6.20 ± 0.02 ^d^	6.00 ± 0.05 ^d^
+	–	2.96 ± 0.04 ^a^	3.09 ± 0.07 ^a^	4.00 ± 0.07 ^b^	4.21 ± 0.03 ^b^	4.79 ± 0.05 ^b^	5.34 ± 0.03^c^	5.10 ± 0.02 ^c^	4.65 ± 0.05 ^c^
+	+	2.95 ± 0.08 ^a^	3.24 ± 0.07 ^a^	4.12 ± 0.07 ^b^	4.51 ± 0.03 ^b^	5.00 ± 0.01 ^c^	6.40 ± 0.02 ^d^	5.64 ± 0.02 ^c^	5.45 ± 0.03 ^c^

P = Probiotic bacteria, *Lactobacillus casei *10^8^-10^9^ CFU per mL, S = Starter.

**abcde:** Means followed by the same letter are not significantly different (*p* > 0.05).

**Table 2:** Mean ± SD ratings for the acceptability of Iranian white cheese formulated with starter and *L. acidophilus* (probiotic).

**Starter**	**Probiotic**	**Mean rating ±SD**
–	–	6.92 ± 0.00^a^
+	–	7.78 ± 0.40^b^
–	+	8.07 ± 1.46^b^
+	+	8.79 ± 1.51^c^

abc Means followed by the same letter are not significantly different (*p* > 0.05).

In all cases, at end period storage of cheese, *L. acidophilus* count was more than 10^6^ CFU per g, Assessment of the organoleptic characteristics in various treatments of Iranian white cheese is shown in table 2. The highest and lowest sensory acceptability are for white cheese containing *L. acidophilus* with starter and white cheese without starter and probiotic bacteria, respectively. Values of pH during the cheese ripening period have declined significantly (Table 3) (*p *< 0.05). 

**Table 3:** Mean ± SD ratings for the acceptability of Iranian white cheese formulated with starter and *L. acidophilus* (Probiotic).

	Samples	**Ripening period (days)**					
		1	7	15	30	45	60
**pH changes**	C	5.32^a^	4.90^b^	4.61 ^c^	4.56 ^c^	4.49 ^c^	4.59 ^c^
	L	5.22 ^a^	4.79 ^b^	4.15 ^c^	4.00 ^c^	3.92 ^c^	3.97 ^c^
	LC	5.43 ^a^	4.85 ^b^	4.36^c^	4.22 ^c^	4.19 ^c^	4.21 ^c^

C = Control cheese, L = Probiotic cheese without starter, LC = Probiotic cheese whit starter.

abc Means followed by the same letter are not significantly different (*p* > 0.05).

## Discussion

*Listeria monocytogenes* survives during various stages of production and storage of dairy products, for example yogurt and cheese, produced with starter cultures.^11^ According to a study, 10 cases from 61 cases of disease incidence resulting from consuming dairy products were due to *L. monocytogenes* and among these products, 32.8% were prepared from pasteurized milk.^11^ Presence of these pathogenic bacteria in fermentative dairy products is very likely due to its adaptability with acidic conditions.^12^ Studies conducted on cheese samples produced from milk contaminated with *L. monocytogenes* showed that the behavior of this bacteria (growth, survival and inhibition) in cheese is essentially dependent upon factors such as starter nature and activity, reduced values of pH, temperature and moisture content during various stages of production, ripening and storage.^13^ In this research, during the cheese ripening period pH values have declined significantly (*p < *0.05). Values of pH on the first day of production in all samples were in a statistical level. Reduction in pH values during storage of probiotic cheese samples without starter was the most and was lowest in the control cheese samples. Results of this study are in line with the results of other researcher.^14^^,^^15^ Values of pH decrease during storage due to different amounts of acidifying function of probiotic strains.^14^ They also showed that during ripening of probiotic cheddar cheese at 4 ˚C, the acetic acid concentration increased. In Cheddar cheese containing *Lactobacillus casei, *level of acetic acid at the end ripening period was 0.07%.^14^ In the present study, the highest amount of decrease in *L. monocytogenes* was in probiotic cheese without starter followed by probiotic cheese with starter. This finding is consistent with other reports.^13^^,^^16^^,^^17^ In ripening stage of Colby cheese *L. monocytogenes* counts were gradually decreased from 3.5 - 1.5 Log per gram cheese.^16^ While in blue cheese, after a short time from its production and early stages of ripening, bacterial count decreased and then remained at a fixed amount.^18^ During production of Camembert cheese, *L. monocytogenes* count was increased to 10 times of its primary count and decreased during first 18 days of ripening period and finally its growth was inhibited due to increased acidity.^19^ Regarding the antimicrobial activity of the starter producing nisin against *L. monocytogenes* in Manchego cheese prepared from goat raw milk, showed that in treatments having starter producing nisin, *Listeria* decrease was 4.62 Log at the end of cheese storage and in treatment having starter without production of nisin, it was 1.66 Log and in the treatment combining 2 previous ones, it was 3.31 Log per gram of cheese.^17^

Evaluation of durability of *L. monocytogenes* in various stages of production, ripening and storage of soft lactic cheese produced from cow milk contaminated with this bacteria showed that during ripening stage, *L. monocytogenes* count decreased but could be isolated.^20^ In this study, *L. monocytogenes* count decreased at the end of ripening period but it still survived in cheese. This finding is consistent with other reports.^18^^,^^20^ Lactic acid bacteria producing bacteriocin-s are extensively used in production of fermentative foods in order to improve sensory characteristics and also preventing food perishing. In this regard, *Lactobacilli* have drawn great attention because of having characteristics such as host health promotion.^21^ In order to use probiotic bacteria in functional foods, these organisms should survive passing in digestive system and also should have the ability of reproduction. In this regards, Lactobacilli are probiotic agents which have the ability to remain in digestive system and also improve its microflora balance.^21^ *Lactobacillus casei *in combination with commercial starters being used in dairy product industry have shown antimicrobial and antitumor characteristics and simulate immunity system.^22 ^Administration of *L. casei* to rats infected with *L. monocytogenes* led to increase in immune cells.^23^ Administration of this probiotic bacteria to mice infected with *E. coli,* prevented the growth and infectivity of this pathogenic bacteria.^24^^,^^25^ Decease in* L. acidophilus* count during initial 15 days of white cheese ripening was higher compared to other days and the reason for this fact was reported as decrease in moisture content, increase in salt amount and decrease in storage temperature.^25^ High levels of salt and low relative pH of Iranian white cheese may cause a reduction in the number of probiotic bacteria during storage of the cheese.^26^^,^^27^ Though in the present study, *L. acidophilus* count decreased during Iranian white cheese ripening period but its amount did not decrease to less than 10^6^ CFU per g at the end of ripening and storage period.

In conclusion, decrease in *L. monocytogenes *count during ripening and storage periods of probiotic Iranian white cheese can be due to combined effect of pH decreased and antimicrobial activity of starter and probiotic bacteria used in this study. But the environment conditions were not sufficient enough to eliminate this pathogen in white cheese completely. In order to ensure health and microbial resistance of fermentative dairy products, food industries should not rely on pasteurization and fermentation. Permitted additives should also be used during various stages of production and storage in order to create desirable conditions. Durability of the probiotic bacteria examined in the present study was in the range required for exerting useful effects on health. Also the mentioned bacteria showed positive effects on cheese sensory characteristics. Thus Iranian white cheese is very suitable as a food carrying probiotic bacteria.
